# Association between creatinine clearance and lumbar bone mineral density in Chinese patients with osteoporotic fractures: a retrospective cross-sectional study

**DOI:** 10.3389/fendo.2026.1696728

**Published:** 2026-01-28

**Authors:** Ming Su, Peng Zhou, Li-long Feng, Hui-qiang Shan, Min-zhe Xu, Ya-qin Gong, Jian Jin, Wen-bin Hu, Ke Lu, Chong Li, Yi Yin

**Affiliations:** 1 Department of Orthopedics, Affiliated Kunshan Hospital of Jiangsu University, Suzhou, Jiangsu, China; 2 Kunshan Biomedical Big Data Innovation Application Laboratory, Suzhou, Jiangsu, China; 3 Information Department, Affiliated Kunshan Hospital of Jiangsu University, Suzhou, Jiangsu, China; 4 Kunshan Municipal Health and Family Planning Information Center, Suzhou, Jiangsu, China; 5 Chronic Disease Department, Kunshan Center for Disease Control and Prevention, Suzhou, Jiangsu, China

**Keywords:** creatinine clearance, elderly osteoporosis, lumbar BMD, osteoporotic fractures, renal function

## Abstract

**Background:**

Creatinine clearance (CCR) is an important marker of renal function. The association between CCR and lumbar bone mineral density (BMD) in patients with osteoporotic fractures (OPFs) remains unclear. With the increasing prevalence of OPFs in aging populations and the complications associated with renal impairment, understanding this relationship is essential for optimizing patient outcomes.

**Methods:**

A total of 1,313 patients with OPFs (aged ≥50 years) were retrospectively analyzed. Patient CCR, lumbar BMD, and relevant clinical data were collected. Lumbar BMD (g/cm^2^) served as the outcome variable, while baseline CCR was the exposure variable. Multivariate analyses were performed with adjustments for multiple covariates.

**Results:**

In fully adjusted multivariate regression analysis, baseline CCR showed a significant positive correlation with lumbar BMD (β = 0.16; 95% CI: 0.13–0.18; *P* < 0.01). A threshold effect was identified in the CCR–BMD relationship, characterized by an inflection point at approximately 130 mL/min CCR. Below this threshold, higher CCR was associated with increased lumbar BMD. Above 130 mL/min, further increases in CCR were not associated with further BMD gains (β = –0.05, *P* = 0.18). Mean lumbar BMD values increased across CCR tertiles (0.69 ± 0.13, 0.73 ± 0.14, and 0.81 ± 0.15 g/cm^2^; *P* < 0.01).

**Conclusion:**

CCR was positively associated with lumbar BMD among elderly patients with OPFs, showing a non-linear relationship characterized by a CCR threshold (~130 mL/min). These findings highlight the potential importance of renal function assessment in osteoporosis risk evaluation and fracture management.

## Introduction

Osteoporotic fractures (OPFs) represent a significant public health challenge among elderly populations due to their association with significantly increased morbidity and mortality (1–3). Typically, OPFs occur in older individuals following osteoporosis-related reductions in bone mineral density (BMD) and bone strength, predisposing them to fractures at common sites such as the hip, spine, and wrist (4–7). These fractures often result in prolonged hospitalization, reduced quality of life, and extensive healthcare expenditures. Long-term complications of OPFs include permanent disability, loss of independence, and an increased risk of subsequent fractures (8, 9). Collectively, these impacts impose a significant socioeconomic burden on global healthcare systems. Therefore, identifying and managing risk factors influencing outcomes among patients with OPFs is important to mitigate these adverse effects (10).

Creatinine clearance (CCR) is a commonly employed measure of renal function and is influenced by skeletal muscle mass. Adequate kidney function plays a critical role in bone health through the regulation of calcium-phosphorus homeostasis and activation of vitamin D (11–13). Impaired renal function disrupts these processes and is associated with adverse changes in bone metabolism. Clinical studies have reported that patients with chronic kidney disease (CKD) frequently exhibit reduced BMD, with severity proportional to the decrease in renal function (14). Lower CCR values (or reduced serum creatinine in patients with low muscle mass) have been associated with reduced BMD and increased skeletal fragility (11, 12). These findings suggest that deteriorating renal function may exacerbate osteoporosis and elevate fracture risk.

The precise mechanisms relating renal function and BMD remain incompletely understood. However, one hypothesis is that muscle-bone interactions, potentially mediated by muscle-derived signaling molecules (myokines), may contribute to this relationship (15, 16). Sarcopenia, characterized by loss of muscle mass, frequently overlaps with declining renal function and has been associated with increased osteoporosis prevalence among the elderly. This muscle–kidney–bone interaction may partially explain the observed association between CCR and BMD. Elucidating these relationships may increase the development of comprehensive strategies to reduce fracture risk within this population (17).

Although previous studies have reported associations between renal function and bone health in certain populations, findings have been inconsistent across cohorts and clinical contexts. For example, while Kaji et al. (18) found that mild renal dysfunction was independently associated with lower BMD and vertebral fractures in postmenopausal women, Jassal et al. (19) observed no significant relationship between renal function and BMD or fracture risk in community-dwelling older adults. Moreover, the specific association between CCR and lumbar BMD in patients who have already sustained OPFs remains poorly characterized. Therefore, this study aimed to investigate the relationship between CCR and lumbar BMD among patients aged ≥50 years diagnosed with OPFs. The goal was to extend current knowledge by clarifying how variations in renal function correlate with lumbar BMD, thus informing clinical strategies for osteoporosis prevention and fracture risk management in affected patient populations.

## Materials and methods

### Data origin

A retrospective cross-sectional study was conducted using electronic medical records of patients aged ≥50 years who were newly diagnosed with OPFs requiring surgical hospitalization at Kunshan First People’s Hospital affiliated with Jiangsu University between December 2018 and December 2023. To identify only incident fractures, patients with any documented fractures in the five years before the index fracture were excluded. Data from patients admitted during this period were consecutively collected and reviewed. Fractures involving the wrist, proximal humerus, hip, and vertebrae were identified using ICD-10 codes (S22, S32, S42, S52, S72), consistent with established definitions of OPFs used in previous epidemiological studies. Clinical data systematically extracted from patient records included demographic information (age, sex, weight); laboratory variables, including coagulation indices (prothrombin time [PT], activated partial thromboplastin time [APTT]), platelet count, hemoglobin, serum albumin, serum calcium, neutrophil count, lymphocyte count, monocyte count, serum potassium, uric acid, and CCR; clinical characteristics such as the American Society of Anesthesiologists (ASA) physical status classification (grade 1, 2, or ≥3); and medical history, including hypertension status, smoking status, and Charlson Comorbidity Index (CCI) category. All laboratory analyses were performed according to standardized procedures in an accredited clinical laboratory, and all clinical evaluations were completed within three days of hospital admission. A consecutive enrollment approach was used to minimize selection bias and ensure the sample’s representativeness.

### Ethical statement

The study protocol was approved by the Ethics Committee of the Affiliated Kunshan Hospital of Jiangsu University (Suzhou, China; approval number: 2024-03-053-H00-K01). All procedures were conducted following the ethical standards outlined in the Declaration of Helsinki. Patient confidentiality and data privacy were strictly maintained throughout the analysis.

### Study design and participants

**Figure 1:**
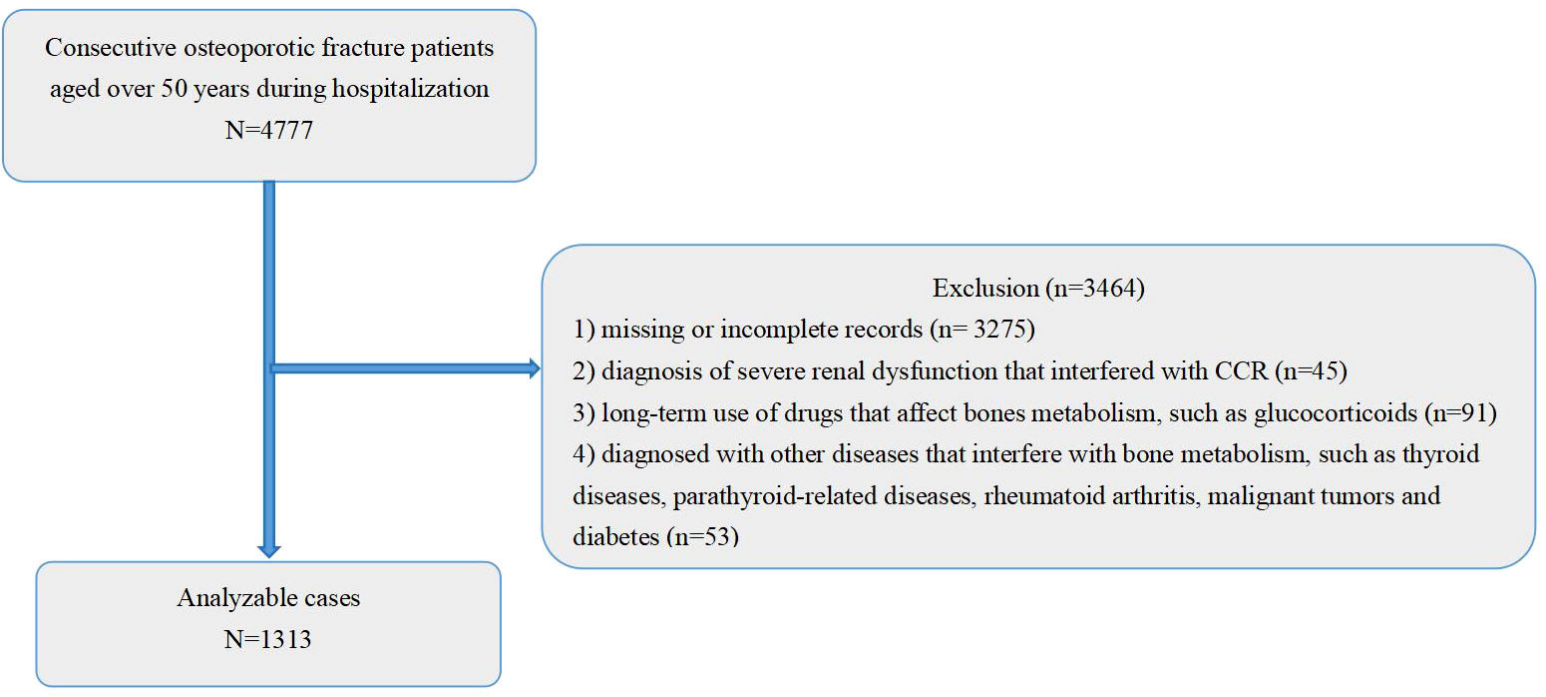
A schematic representation of the study design. CCR, Creatinine clearance.

This retrospective cross-sectional study included patients aged ≥50 years with newly diagnosed OPFs who were hospitalized between January 1, 2018, and December 31, 2023, at the Affiliated Kunshan Hospital of Jiangsu University (Suzhou, China). OPFs were defined by standard criteria as (1) a fragility fracture occurring without other metabolic bone disease and/or (2) a BMD T-score ≤ –2.5 even in the absence of fracture (20). All patients had routine blood tests upon admission. A total of 4,777 patients aged ≥50 years with incident OPFs were initially screened. After applying the exclusion criteria, 1,313 eligible patients were enrolled in the final analysis. Exclusion criteria included: (1) missing or incomplete medical records (n = 3,275); (2) severe renal dysfunction potentially interfering with CCR measurements (n = 45) (21); (3) long-term use of medications known to significantly affect bone metabolism, such as glucocorticoids (n = 91) (22, 23); and (4) comorbid conditions affecting bone metabolism, such as thyroid or parathyroid disorders, rheumatoid arthritis, malignancy, or uncontrolled diabetes (n = 53) (22–24). Patients meeting inclusion criteria were consecutively enrolled to minimize selection bias, thereby ensuring the representativeness of the sample population (**Figure 1**).

### Exposure and outcome variables

CCR was calculated using the Cockcroft–Gault formula: CCR = (140−age (years))×body weight (kg)/72×plasma creatinine (mg/dL), with a multiplication factor of 0.85 applied for females (25, 26). The calculated CCR (mL/min) served as the primary exposure variable. Lumbar BMD, the outcome variable, was measured using dual-energy X-ray absorptiometry (DXA) with a Hologic Discovery Wi bone densitometer (Hologic Inc., USA). Fasting blood samples were collected within 24 hours of admission to measure plasma creatinine for CCR calculation. All measurements were conducted by trained personnel following standardized protocols. For patients with a lumbar vertebral fracture, lumbar BMD was calculated as the mean BMD of the intact lumbar vertebrae to minimize bias from the fractured segment.

### Covariate variables

The covariates in this study included laboratory parameters (coagulation measures, blood counts, and metabolic measures), clinical indices (comorbidity indices), lifestyle factors (smoking), and medical history (hypertension). All laboratory tests were performed using standardized equipment. PT and APTT were measured using an automated coagulation analyzer (Sysmex CN-6000). Complete blood counts, including platelet and differential counts for neutrophils, lymphocytes, and monocytes, were obtained using a Sysmex XN-10 hematology analyzer. Hemoglobin was measured using the sodium lauryl sulfate hemoglobin (SLS-Hb) method on the XN-10 analyzer. Serum albumin, potassium, and uric acid were measured using a Beckman AU5800 clinical chemistry analyzer with standard colorimetric and enzymatic assays. For clinical indices, an anesthesiologist assigned the ASA physical status category to rate each patient’s preoperative health, with higher categories indicating greater systemic risk (27). The Charlson Comorbidity Index was calculated by summing weighted scores assigned to comorbid conditions, with higher scores indicating more significant comorbidity burden and increased 10-year mortality risk (28, 29). The ASA category (1, 2, or ≥3) and the CCI score category were recorded for all patients. All clinical and laboratory assessments were completed within the first 72 hours of hospitalization.

### Statistical analyses

Baseline patient characteristics, stratified by CCR tertiles, were summarized using mean standard deviation (SD) for continuous variables and frequencies (percentages) for categorical variables (**Table 1**). Group comparisons were conducted using the independent Student’s t-test for continuous variables with approximate normality and the Mann–Whitney U test for non-normally distributed continuous variables. Categorical variables were compared using the chi-square test, with Fisher’s exact test applied when expected cell counts were small. Multicollinearity among covariates was assessed using variance inflation factors (VIF) before regression modeling. Covariates were selected for adjustment based on their confounding potential for multivariable analyses. Variables were considered confounders if they modified the CCR–BMD association by more than 10% when added to a basic model (CCR and BMD only) or demonstrated a univariate association with BMD at *P* < 0.1. Three models were constructed for regression analysis. Model 1 included CCR and BMD without adjustment. Model 2 included adjustments for key laboratory covariates: PT, APTT, platelet count, hemoglobin, albumin, calcium, neutrophil count, lymphocyte count, monocyte count, and potassium. Model 3 included all covariates in Model 2, along with uric acid, ASA category, hypertension, smoking status, and CCI category. Subgroup analyses were conducted to assess the robustness of the CCR–BMD association across patient subgroups. Patients were stratified by factors such as sex and hypertension status, and interaction terms were used to evaluate whether the CCR–BMD association differed significantly between subgroups. Smoothing spline plots were used to explore non-linear associations between CCR and BMD. All statistical analyses were conducted using EmpowerStats (X&Y Solutions, Boston, MA, USA) and R software. A two-tailed *P* < 0.05 was considered statistically significant.

**Table 1:** Characteristic of study participants.

Characteristics	Total mean ± SD	T1(<59.99 ml/min) mean ± SD	T2(59.99-88.08 ml/min) mean ± SD	T3(>88.08 ml/min) mean ± SD	*P*-value*
N	1313	438	437	438	
Gender, N (%)					<0.01
female	993 (75.63%)	388 (88.58%)	349 (79.86%)	256 (58.45%)	
male	320 (24.37%)	50 (11.42%)	88 (20.14%)	182 (41.55%)	
Age, years	68.78 ± 10.67	76.15 ± 9.74	67.84 ± 9.11	62.36 ± 8.21	<0.01
CCR, ml/min	78.13 ± 35.15				
CCR, per 100 ml/min increase	0.78 ± 0.35	0.44 ± 0.11	0.74 ± 0.08	1.17 ± 0.28	<0.01
Lumbar BMD, g/cm2	0.74 ± 0.14	0.69 ± 0.13	0.73 ± 0.14	0.81 ± 0.15	<0.01
Lumbar BMD T-score	−2.17 ± 1.17	−2.58 ± 1.08	−2.25 ± 1.17	−1.58 ± 1.25	<0.01
Femoral neck BMD T-score	-4.00 ± 0.92	-4.33 ± 0.75	-3.92 ± 0.75	-3.58 ± 0.92	<0.01
Hip BMD T-score	-2.50 ± 1.17	-3.08 ± 1.00	-2.42 ± 1.00	-1.83 ± 1.08	<0.01
PT, s	11.83 ± 1.46	11.88 ± 1.47	11.68 ± 1.35	11.93 ± 1.53	0.01
APTT, s	28.24 ± 4.13	28.89 ± 4.37	28.04 ± 3.94	27.80 ± 3.98	<0.01
Platelet, ×10^9^/L	177.36 ± 62.37	168.79 ± 60.35	178.30 ± 63.32	184.98 ± 62.47	<0.01
Hemoglobin, g/L	123.14 ± 18.69	120.21 ± 21.10	125.49 ± 17.53	123.71 ± 16.81	<0.01
Albumin, g/L	39.57 ± 4.34	38.96 ± 4.91	39.99 ± 4.02	39.75 ± 3.99	<0.01
Calcium, mmol/L	2.19 ± 0.13	2.19 ± 0.13	2.20 ± 0.13	2.18 ± 0.14	0.13
Neutrophil, ×10^9^/L	6.55 ± 3.23	6.74 ± 3.36	6.30 ± 3.13	6.60 ± 3.18	0.05
Lymphocyte, ×10^9^/L	1.23 ± 0.55	1.19 ± 0.53	1.28 ± 0.58	1.23 ± 0.53	0.07
Monocyte, ×10^9^/L	0.50 ± 0.25	0.52 ± 0.26	0.49 ± 0.24	0.49 ± 0.23	0.05
Potassium, mmol/L	3.86 ± 0.44	3.94 ± 0.46	3.83 ± 0.42	3.80 ± 0.42	<0.01
UA, μmol/L	279.57 ± 92.28	313.38 ± 107.50	270.30 ± 81.63	255.07 ± 74.40	<0.01
25(OH)D levels, ng/mL	19.18 ± 7.67	17.68 ± 8.71	19.70 ± 8.12	19.75 ± 6.43	0.28
Apolipoprotein A, g/L	1.17 ± 0.22	1.11 ± 0.19	1.17 ± 0.21	1.23 ± 0.24	<0.01
Apolipoprotein B, g/L	0.78 ± 0.22	0.74 ± 0.22	0.79 ± 0.21	0.81 ± 0.22	<0.01
Homocysteine, μmol/L	14.33 ± 9.60	16.65 ± 11.41	13.52 ± 8.66	12.90 ± 8.05	<0.01
High-density lipoprotein, mmol/L	1.31 ± 0.32	1.26 ± 0.30	1.29 ± 0.31	1.38 ± 0.34	<0.01
Low-density lipoprotein, mmol/L	2.52 ± 0.76	2.41 ± 0.72	2.61 ± 0.79	2.55 ± 0.77	0.01
Total cholesterol, mmol/L	4.21 ± 0.93	4.03 ± 0.92	4.27 ± 0.91	4.31 ± 0.95	<0.01
Triglyceride, mmol/L	1.23 ± 0.80	1.20 ± 0.86	1.30 ± 0.86	1.19 ± 0.66	0.19
ASA, N (%)					<0.01
1	225 (17.14%)	50 (11.42%)	86 (19.68%)	89 (20.32%)	
2	829 (63.14%)	234 (53.42%)	286 (65.45%)	309 (70.55%)	
≥3	259 (19.73%)	154 (35.16%)	65 (14.87%)	40 (9.13%)	
Hypertension, N (%)					<0.01
no	1170 (89.11%)	374 (85.39%)	391 (89.47%)	405 (92.47%)	
yes	143 (10.89%)	64 (14.61%)	46 (10.53%)	33 (7.53%)	
Smoking, N (%)					<0.01
no	1177 (92.97%)	421 (98.36%)	399 (95.00%)	357 (85.41%)	
yes	89 (7.03%)	7 (1.64%)	21 (5.00%)	61 (14.59%)	
CCI score category, N (%)					0.01
0	1235 (94.06%)	396 (90.41%)	419 (95.88%)	420 (95.89%)	
1	69 (5.26%)	36 (8.22%)	16 (3.66%)	17 (3.88%)	
≥2	9 (0.69%)	6 (1.37%)	2 (0.46%)	1 (0.23%)	

CCR, creatinine clearance; T1, first tertile; T2, second tertile; T3, third tertile; SD, standard deviation; BMD, bone mineral density; PT, Prothrombin Time; APTT, Activated Partial Thromboplastin Time; UA, uric acid; 25(OH)D, 25-hydroxy vitamin D; ASA, American Society of Anesthesiologists; CCI, Charlson Comorbidity Index.

*Kruskal-Wallisrank test for continuous variables, Fisher exact for categorical variables with expects<10.

## Results

### Baseline study population characteristics

A total of 1,313 patients met the inclusion criteria for analysis. The mean patient age was 68.78 ± 10.67 years, and 24.37% were male. The overall mean lumbar BMD was 0.74 ± 0.14 g/cm^2^ and the mean CCR was 78.13 ± 35.15 mL/min. Patients were stratified into three CCR tertiles: <59.99 mL/min (T1), 59.99–88.08 mL/min (T2), and >88.08 mL/min (T3). **Table 1** summarizes the baseline characteristics by CCR tertile. Several characteristics differed significantly across the CCR groups, including age, sex distribution, lumbar BMD, PT, APTT, platelet count, hemoglobin, albumin, calcium, neutrophil count, monocyte count, potassium, uric acid, lipid profile (apolipoproteins, homocysteine, and cholesterol levels), ASA category, hypertension prevalence, smoking status, and CCI category. Lumbar BMD increased progressively with higher CCR: the mean BMD in T1, T2, and T3 was 0.69 ± 0.13, 0.73 ± 0.14, and 0.81 ± 0.15 g/cm^2^, respectively (overall *P* < 0.01 for trend). Patients with better renal function had higher lumbar BMD.

### Association between CCR and lumbar BMD

The association between CCR and lumbar BMD was analyzed using three models (**Table 2**). In the unadjusted model (Model 1), CCR showed a significant positive association with BMD (β = 0.14, 95% CI: 0.12–0.16; *P* < 0.01). After adjustment for laboratory covariates in Model 2 (PT, APTT, platelets, hemoglobin, albumin, calcium, neutrophils, lymphocytes, monocytes, and potassium), the association remained unchanged (β = 0.15, 95% CI: 0.13–0.17; *P* < 0.01). In the fully adjusted Model 3, which included all Model 2 covariates plus uric acid, ASA category, hypertension, smoking, and CCI category, CCR remained positively associated with lumbar BMD (β = 0.16, 95% CI: 0.13–0.18; *P* < 0.01). In the fully adjusted model, the difference in lumbar BMD between CCR tertiles was significant: lumbar BMD in the middle tertile (T2) was 0.06 g/cm^2^ higher than in the lowest tertile (T1), and BMD in the highest tertile (T3) was 0.13 g/cm^2^ higher than in T1. This trend of increasing BMD with higher CCR was observed in all models.

**Table 2:** Association between CCR and lumbar BMD in different models.

Exposure	Model 1^a^ N = 1313 β (95% CI) *P-*value	Model 2^b^ N = 1275 β (95% CI) *P-*value	Model 3^c^ N = 1228 β (95% CI) *P-*value
CCR per 100 ml/min increase	0.14 (0.12, 0.16) <0.01	0.15 (0.13, 0.17) <0.01	0.16 (0.13, 0.18) <0.01
CCR tertile			
T1(<59.99 ml/min)	Reference	Reference	Reference
T2(59.99-88.08 ml/min)	0.05 (0.03, 0.06) <0.01	0.05 (0.03, 0.07) <0.01	0.06 (0.04, 0.08) <0.01
T3(>88.08 ml/min)	0.12 (0.10, 0.14) <0.01	0.13 (0.11, 0.15) <0.01	0.13 (0.11, 0.15) <0.01

a No adjustment.

b Adjusted for PT, APTT, platelet counts, hemoglobin, albumin, calcium, neutrophils, lymphocytes, monocytes, and potassium.

c Adjusted for PT, APTT, platelet counts, hemoglobin, albumin, calcium, neutrophils, lymphocytes, monocytes, potassium, UA, ASA category, hypertension, smoking and CCI score category.

### Subgroup analyses

Subgroup analyses were conducted to determine whether the CCR–BMD association remained consistent across patient subgroups stratified by variables such as ASA category and hypertension status. The positive association between CCR and BMD was observed in all subgroups, with no significant interaction effects, indicating no significant variation in the association across subgroups. The association between higher CCR and higher BMD remained strong and was consistent across all subgroups (**Table 3**).

**Table 3:** Subgroup analysis between CCR and lumbar BMD.

Subgroup	N	Lumbar BMD^a^ β (95% CI) *P*-value
X=CCR, per 100 ml/min increase		
PT, s		
9.10-11.60	652	0.20 (0.16, 0.23) <0.01
11.70-33.90	653	0.13 (0.09, 0.16) <0.01
APTT, s		
18.70-27.70	645	0.17 (0.13, 0.20) <0.01
27.80-67.30	660	0.15 (0.11, 0.19) <0.01
Platelet, ×10^9^/L		
10.00-169.00	655	0.19 (0.15, 0.23) <0.01
170.00-515.00	657	0.14 (0.11, 0.17) <0.01
Hemoglobin, g/L		
43.00-124.00	656	0.15 (0.11, 0.18) <0.01
125.00-171.00	656	0.18 (0.14, 0.21) <0.01
Albumin, g/L		
14.50-39.70	638	0.17 (0.14, 0.21) <0.01
39.80-52.50	645	0.14 (0.11, 0.18) <0.01
Calcium, mmol/L		
0.74-2.18	631	0.18 (0.14, 0.21) <0.01
2.19-2.91	679	0.15 (0.11, 0.18) <0.01
Neutrophil, ×10^9^/L		
1.10-5.98	656	0.18 (0.14, 0.21) <0.01
5.99-29.16	656	0.15 (0.11, 0.18) <0.01
Lymphocyte, ×10^9^/L		
0.10-1.15	654	0.16 (0.13, 0.20) <0.01
1.16-4.76	658	0.16 (0.12, 0.19) <0.01
Monocyte, ×10^9^/L		
0.00-0.48	649	0.16 (0.13, 0.19) <0.01
0.49-2.90	663	0.16 (0.12, 0.20) <0.01
Potassium, mmol/L		
2.02-3.84	651	0.16 (0.12, 0.19) <0.01
3.85-6.62	659	0.16 (0.12, 0.20) <0.01
UA, μmol/L		
79.00-267.20	653	0.16 (0.12, 0.19) <0.01
268.00-997.00	658	0.15 (0.12, 0.19) <0.01
ASA, N (%)		
1	225	0.15 (0.09, 0.20) <0.01
2	829	0.16 (0.13, 0.19) <0.01
≥3	259	0.16 (0.09, 0.22) <0.01
Hypertension, N (%)		
no	1170	0.16 (0.14, 0.19) <0.01
yes	143	0.12 (0.02, 0.21) 0.01
Smoking, N (%)		
no	1177	0.17 (0.14, 0.20) <0.01
yes	89	0.09 (-0.01, 0.19) 0.08
CCI score category, N (%)		
0	1235	0.16 (0.13, 0.18) <0.01
1	69	0.21 (0.09, 0.34) <0.01
≥2	9	NA

a Patients were stratified based on PT, APTT, platelet counts, hemoglobin, albumin, calcium, neutrophils, lymphocytes, monocytes, potassium, UA, ASA category, hypertension, smoking and CCI score category, and additional covariates not included in the stratification were adjusted for in the analysis.

### Spline smoothing plot and threshold analyses

**Figure 2:**
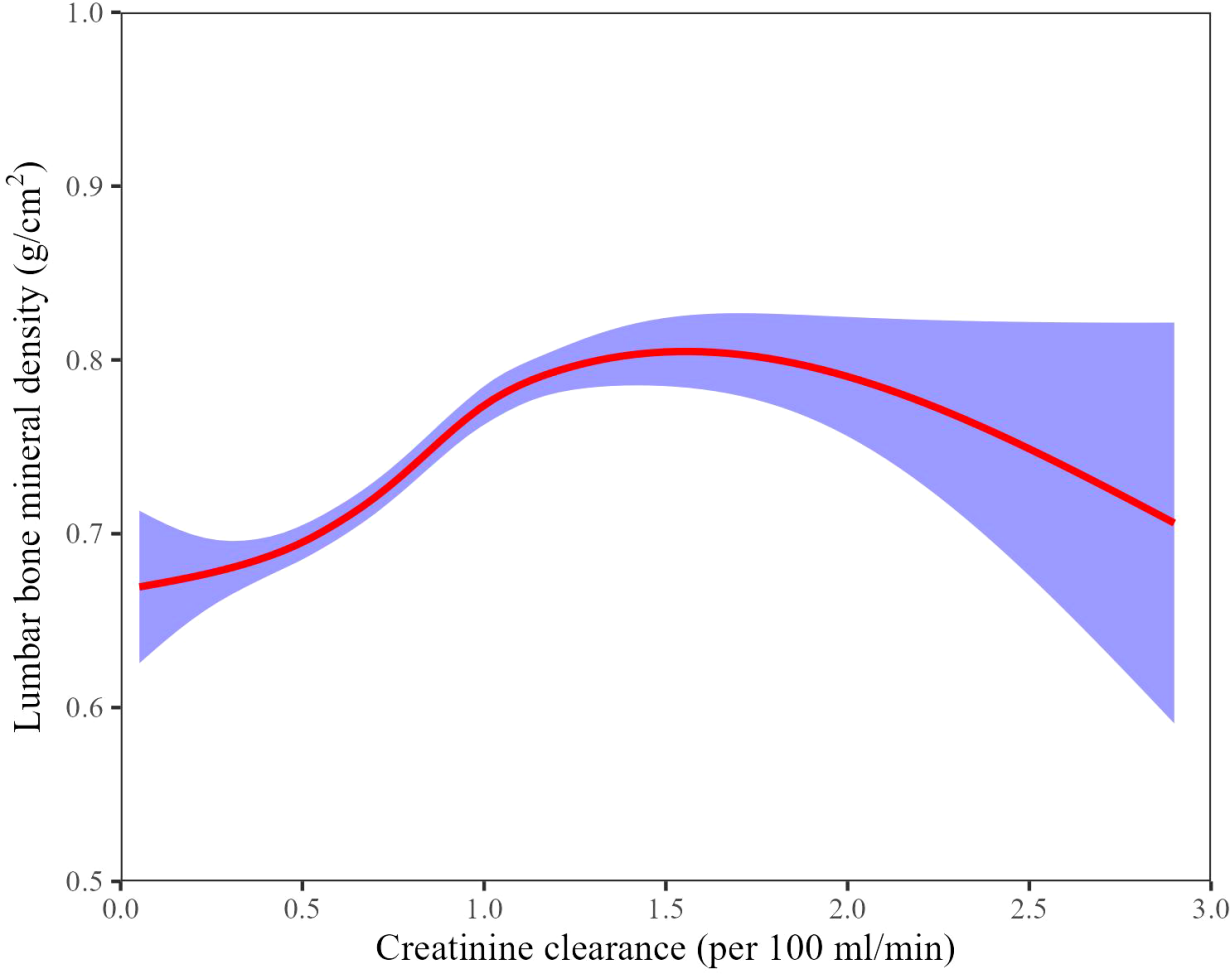
Adjusted smoothed curves corresponding to the relationship between the CCR and lumbar BMD among inpatients with OPFs. The adjusted factors were PT, APTT, platelet counts, hemoglobin, albumin, calcium, neutrophils, lymphocytes, monocytes, potassium, UA, ASA category, hypertension, smoking and CCI score category. CCR, creatinine clearance; BMD, bone mineral density; OPFs, Osteoporotic Fractures; PT, Prothrombin Time; APTT, Activated Partial Thromboplastin Time; UA, uric acid; ASA, American Society of Anesthesiologists; CCI, Charlson Comorbidity Index.

A smoothed spline plot indicated a non-linear relationship between CCR and lumbar BMD in the fully adjusted model. A threshold effect was assessed using a two-piece linear regression. The likelihood ratio test indicated a significant non-linear association (*P* < 0.01), and an inflection point for CCR was identified at approximately 130 mL/min (**Figure 2**; **Table 4**). Below 130 mL/min, CCR exhibited a significant positive slope with BMD (β = 0.21; 95% CI: 0.18–0.24; *P* < 0.01). The slope was near zero above the 130 mL/min threshold and not statistically significant (β = −0.05; 95% CI: −0.13 to 0.02; *P* = 0.18). In other words, lumbar BMD increased with CCR up to approximately 130 mL/min, beyond which further increases in CCR were not associated with additional BMD gains.

**Table 4:** Threshold analyses examining the relationship between CCR and lumbar BMD.

Model 3^a^ Lumbar BMD	β (95% CI) P-value
Model A^b^	
One line slope	0.16 (0.13, 0.18) <0.01
Model B^c^	
CCR turning point (K), per 100 ml/min	1.30
< K	0.21 (0.18, 0.24) <0.01
> K	-0.05 (-0.13, 0.02) 0.18
Slope 2-Slope 1	-0.26 (-0.35, -0.17) <0.01
LRT^d^	<0.01

a Adjusted for PT, APTT, platelet, hemoglobin, albumin, calcium, neutrophil, lymphocyte, monocyte, potassium, UA, ASA category, hypertension, smoking and CCI score category.

b Linear analysis, *P*-value<0.05 indicates a linear relationship.

c Nonlinear analysis.

d *P*-value < 0.05, indicating that Model B is significantly different from Model A, which indicates a nonlinear relationship.

## Discussion

The study background highlights that CCR is a crucial marker of renal function, yet its relationship with lumbar BMD in elderly patients with OPFs remains unclear. This retrospective cross-sectional study involving 1,313 patients with OPFs identified a significant positive association between CCR and lumbar BMD. Higher CCR, indicative of improved renal function, was associated with increased lumbar BMD. A non-linear pattern was observed, with an apparent CCR threshold of approximately 130 mL/min, beyond which the association plateaued. These findings suggest that good renal function may contribute to BMD preservation, with a plateau effect beyond a certain threshold.

Impaired renal function, such as those observed in patients with CKD, is widely known to be associated with disorders of bone metabolism, including accelerated bone loss, mineralization defects, and osteoporosis (30). Previous studies exploring the relationship between renal function indicators (CCR or glomerular filtration rate) and BMD across different populations have yielded inconsistent results. Some studies reported a positive relationship between CCR and BMD, whereas others found no clear association or observed that patients with moderate CKD could have low BMD even with relatively preserved CCR levels (19, 31, 32). Our findings align with studies indicating a positive association between renal function and bone density, demonstrating that CCR was an independent positive predictor of lumbar BMD (β = 0.16, *P* < 0.01). A non-linear threshold effect was observed, with an inflection point at approximately 130 mL/min CCR. Below this threshold, the CCR–BMD association was positive, whereas at higher CCR values, the relationship was no longer significant. This threshold finding is novel and suggests that while improved renal function is associated with higher BMD in the suboptimal range, once CCR exceeds approximately 130 mL/min—near the upper physiological range for older adults—further increases in CCR may not translate into additional BMD gains.

This study presents evidence linking renal function to bone health in patients with OPF. The observed positive CCR–BMD association is consistent with the concept that better kidney function is beneficial for bone density. These findings align with previous research. For example, a study found that higher CCR was associated with higher hip BMD in older adults (19). However, findings from previous studies remain inconsistent. Guo et al. (2021) reported that individuals with lower CCR had lower BMD (15), which is consistent with these findings. However, another study reported that even patients with relatively high CCR can experience low BMD and fractures in the setting of CKD (12). Such discrepancies in the literature suggest that the impact of renal function on bone may be influenced by factors such as age, comorbidities, or the presence of CKD-related mineral bone disorder and that the relationship may show non-linearity across different ranges of kidney function. The observed threshold effect, in which no further BMD benefit was observed beyond a certain CCR level, may help explain these discrepancies by indicating a point of renal function beyond which other factors predominate in bone health.

These findings highlight the association between kidney function and bone metabolism. Impaired kidney function can adversely affect bone through multiple biological pathways. In CKD, for example, declining renal function leads to disturbances in calcium and phosphate homeostasis and secondary hyperparathyroidism, which accelerate bone loss and increase fracture risk (30). CCR, as a measure of glomerular filtration and overall renal health, may serve as a proxy for these pathogenic processes, as a lower CCR may reflect reduced renal clearance of phosphorus and decreased activation of vitamin D, leading to higher parathyroid hormone (PTH) levels and consequent bone resorption. In this study, lumbar BMD tended to increase as CCR increased, which is consistent with this mechanism and previous observations that low CCR is associated with low BMD (16). Moreover, CCR is influenced by muscle mass, and muscle health is closely related to bone health (33). Skeletal muscle secretes myokines that have anabolic effects on bone. Sarcopenia (loss of muscle mass), commonly associated with renal impairment and aging, is strongly linked to reduced bone density in the elderly (34). Thus, the CCR–BMD relationship may be explained by muscle mass, as patients with higher CCR often have greater muscle mass, which in turn positively affects bone density. These findings suggest a muscle–kidney–bone axis in which better kidney function and higher muscle mass support higher BMD. These findings support a multifactorial model in which better renal function (higher CCR) is associated with higher BMD, likely through direct renal effects on mineral metabolism and indirect effects *via* muscle. Emerging evidence further supports this interpretation, suggesting that the association between renal function and BMD may, at least in part, be mediated by muscle–bone interactions characterized by shared endocrine and metabolic pathways. Recent studies have described a bidirectional relationship between skeletal muscle and bone health, especially in patients with chronic kidney disease, where the coexistence of sarcopenia and low BMD—referred to as *osteosarcopenia*—is increasingly recognized as a distinct clinical entity (35, 36) . Mechanistically, several pathways may underlie this link: (1) endocrine and paracrine cross-talk between muscle-derived myokines (e.g., myostatin) and bone-derived osteokines (e.g., osteocalcin) jointly regulate muscle and bone metabolism; (2) chronic inflammation and oxidative stress associated with renal dysfunction induce both muscle catabolism and bone resorption; (3) disturbances in mineral and hormonal homeostasis, including secondary hyperparathyroidism, vitamin D deficiency, and FGF-23/Klotho axis dysregulation, impair both bone formation and muscle protein synthesis; and (4) reduced physical activity and protein-energy wasting exacerbate loss of muscle and bone mass. These convergent mechanisms offer a biologically plausible explanation for the observed renal function–BMD association, while underscoring the need for further longitudinal and mechanistic studies to substantiate this pathway.

It is important to acknowledge that this study lacked data on several lifestyle factors that affect BMD, including diet, physical activity, alcohol use, and vitamin D intake. These unmeasured factors may have confounded the relationship between CCR and BMD. For example, dietary calcium and protein intake affect bone density, and physical exercise helps maintain bone mass, while a sedentary lifestyle accelerates bone loss (37, 38). If patients with better renal function were also more likely to have healthier lifestyles, such as higher-quality nutrition and greater physical activity, this could partly account for their higher BMD. Although many clinical covariates were adjusted for, residual confounding by lifestyle factors remains possible. Future studies should include lifestyle and nutritional assessments to determine the extent to which these factors explain the CCR-BMD association.

Further research is needed to validate and expand these findings. Longitudinal studies or randomized trials, such as those investigating interventions that improve renal function or muscle mass, may help establish causality in the CCR–BMD relationship. Studies in larger and more diverse populations, including patients with advanced CKD, are needed, as individuals with severe renal dysfunction were excluded from this study. In patients with CKD, the interplay between kidney function and bone health is often complicated by chronic inflammation and hormonal disturbances, such as elevated FGF23 or PTH levels. Investigating CCR-BMD associations in these settings could clarify whether these findings are generalizable or if other factors influence the effect of CCR in advanced CKD. However, this study suggests that renal function is an important factor in bone health. Therefore, assessing kidney function could increase osteoporosis risk stratification. Patients with reduced CCR may benefit from closer monitoring of BMD and early preventive measures against bone loss. On the other hand, maintaining optimal renal function by managing kidney disease risk factors may contribute to a multifaceted approach to protecting bone health in the elderly.

A key strength of this study is the large sample size and comprehensive adjustment for potential confounders, which increase confidence in the observed association between CCR and BMD. These findings may be generalizable to similar hospitalized osteoporotic fracture populations. However, several limitations must be considered. First, the cross-sectional design limits the inference of causality or temporal direction. CCR and BMD were assessed at a single time point post-fracture; therefore, it is impossible to determine whether lower CCR led to higher bone loss or whether unmeasured factors affected kidney function and bone density. Second, because all participants had an acute fracture, acute-phase changes such as inflammation and immobilization may have affected certain laboratory values and possibly BMD. The impact of the fracture event itself on the measurements could not be fully separated. For example, post-fracture cytokines could temporarily affect bone turnover and renal function. Third, although many variables were adjusted for, residual confounding from genetic or unmeasured environmental factors remains possible, as diet and exercise were not recorded. This study was conducted at a single hospital and included an ethnically homogeneous population (Chinese patients), which may limit generalizability to other populations. Fourth, patients with severe renal dysfunction (advanced CKD) were excluded, and these findings primarily apply to individuals with normal to moderately impaired renal function. The CCR–BMD relationship may differ in stage 4–5 CKD or end-stage renal disease. Finally, although the sample size was relatively large, it may not have been sufficient to detect small effect sizes or interactions in subgroup analyses. Future prospective studies with more diverse populations and interventional designs to modify renal function or bone outcomes are needed to confirm these findings and clarify the underlying mechanisms.

## Conclusion

In summary, this study identified a significant positive association between creatinine clearance and lumbar BMD in older patients with OPFs. Patients with better renal function had higher bone density, suggesting that renal function is an important contributor to bone health. A non-linear relationship was observed, with an inflection point at approximately 130 mL/min CCR, beyond which no further BMD benefit was observed. These findings suggest that kidney function should be considered when evaluating osteoporosis risk and making management decisions. Given the aging population and the increasing incidence of OPFs, an interdisciplinary approach that includes monitoring and optimizing renal health may support bone health. Further research is needed to elucidate the causal pathways and develop targeted interventions that simultaneously address renal and skeletal health in at-risk individuals.

## Data Availability

The raw data supporting the conclusions of this article will be made available by the authors, without undue reservation.
