# Supplementary material for: Association between creatinine clearance and lumbar bone mineral density in Chinese patients with osteoporotic fractures: a retrospective cross-sectional study
Source: Front Endocrinol (Lausanne). 2026 Jan 28;17:1696728. doi: 10.3389/fendo.2026.1696728 (PMC12890644; doi:10.3389/fendo.2026.1696728)
Supplement: Supplementary file 1 [file Table1.doc]

Supplementary Table S1. Variance inflation factors (VIF) for covariates included in the regression model

| Variable | VIF |
| --- | --- |
| CCR (per 100 mL/min increase) | 1.3 |
| PT | 1.4 |
| APTT | 1.3 |
| platelet counts | 1.2 |
| hemoglobin | 1.5 |
| albumin | 2 |
| calcium | 1.5 |
| neutrophils | 1.4 |
| lymphocytes | 1.3 |
| monocytes | 1.4 |
| potassium | 1.1 |
| UA | 1.2 |
| ASA category | 1.1 |
| hypertension | 1.1 |
| smoking | 1.1 |
| CCI score category | 1.1 |
|  |  |

Note: All VIF values were < 5, indicating no evidence of problematic multicollinearity among the included covariates.
